# Supplementary material for: Differential dopaminergic modulation of spontaneous cortico–subthalamic activity in Parkinson’s disease
Source: eLife. 2021 Jun 4;10:e66057. doi: 10.7554/eLife.66057 (PMC8177893; doi:10.7554/eLife.66057)
Supplement: Transparent reporting form [file elife-66057-transrepform.docx]

***eLife’s* transparent reporting form**

We encourage authors to provide detailed information *within their submission* to facilitate the interpretation and replication of experiments. Authors can upload supporting documentation to indicate the use of appropriate reporting guidelines for health-related research (see [EQUATOR Network](http://www.equator-network.org/%20)), life science research (see the [BioSharing Information Resource](https://biosharing.org/)), or the [ARRIVE guidelines](http://www.plosbiology.org/article/info:doi/10.1371/journal.pbio.1000412) for reporting work involving animal research. Where applicable, authors should refer to any relevant reporting standards documents in this form.

If you have any questions, please consult our Journal Policies and/or contact us: [editorial@elifesciences.org](mailto:editorial@elifesciences.org).

**Sample-size estimation**

- You should state whether an appropriate sample size was computed when the study was being designed
- You should state the statistical method of sample size computation and any required assumptions
- If no explicit power analysis was used, you should describe how you decided what sample (replicate) size (number) to use

Please outline where this information can be found within the submission (e.g., sections or figure legends), or explain why this information doesn’t apply to your submission:

**We did not do an explicit power analysis. In the original study design we based the number of subjects on the current standards of studies also using local field potential recordings in patients. For the analysis we used then all the available data from the measured Parkinson’s patients in our study. For total number of subjects used for analysis please refer to page 14-15 Section 4 Materials and Methods under the heading Subjects.**

**Replicates**

- You should report how often each experiment was performed
- You should include a definition of biological versus technical replication
- The data obtained should be provided and sufficient information should be provided to indicate the number of independent biological and/or technical replicates
- If you encountered any outliers, you should describe how these were handled
- Criteria for exclusion/inclusion of data should be clearly stated
- High-throughput sequence data should be uploaded before submission, with a private link for reviewers provided (these are available from both GEO and ArrayExpress)

Please outline where this information can be found within the submission (e.g., sections or figure legends), or explain why this information doesn’t apply to your submission:

**Please refer to page 14-16, Section 4 Materials and Methods under the headings Subjects and Pre-processing.**

**Statistical reporting**

- Statistical analysis methods should be described and justified
- Raw data should be presented in figures whenever informative to do so (typically when N per group is less than 10)
- For each experiment, you should identify the statistical tests used, exact values of N, definitions of center, methods of multiple test correction, and dispersion and precision measures (e.g., mean, median, SD, SEM, confidence intervals; and, for the major substantive results, a measure of effect size (e.g., Pearson's r, Cohen's d)
- Report exact p-values wherever possible alongside the summary statistics and 95% confidence intervals. These should be reported for all key questions and not only when the p-value is less than 0.05.

Please outline where this information can be found within the submission (e.g., sections or figure legends), or explain why this information doesn’t apply to your submission:

**Please refer to page numbers 18-22 under the headings Statistical analysis of the states, Intra-medication analysis, Inter-medication analysis and Temporal properties of hidden Markov model states**

(For large datasets, or papers with a very large number of statistical tests, you may upload a single table file with tests, Ns, etc., with reference to sections in the manuscript.)

**Group allocation**

- Indicate how samples were allocated into experimental groups (in the case of clinical studies, please specify allocation to treatment method); if randomization was used, please also state if restricted randomization was applied
- Indicate if masking was used during group allocation, data collection and/or data analysis

Please outline where this information can be found within the submission (e.g., sections or figure legends), or explain why this information doesn’t apply to your submission:

**Please refer to page 14-15 Section 4 Materials and Methods under the heading Subjects.**

**Additional data files (“source data”)**

- We encourage you to upload relevant additional data files, such as numerical data that are represented as a graph in a figure, or as a summary table
- Where provided, these should be in the most useful format, and they can be uploaded as “Source data” files linked to a main figure or table
- Include model definition files including the full list of parameters used
- Include code used for data analysis (e.g., R, MatLab)
- Avoid stating that data files are “available upon request”

Please indicate the figures or tables for which source data files have been provided:

**We have made the code to produce the results and generate the figures available on Github:** <https://github.com/saltwater-tensor/HMM_pipeline>**.**

**However, the raw data cannot be made publicly available due to the European and German data privacy laws. When signing the informed consent forms, our patients consented to using their data for research purposes, but they did not sign a form stating that their data can be shared public, even in anonymized form.  In addition, the MRIs of their heads and brains might make them identifiable. Hence, posting them within a public repository is not possible.**

**The raw data can be requested from the corresponding author for replication of the current results and will then be shared in an anonymized way. We are providing the intermediate Matlab data underlying the figures with our submission.**
